# Macular Neovascularization in Pediatric Patients with Long-Chain 3-Hydroxyacyl-CoA Dehydrogenase Deficiency: A Retrospective Analysis of a Case Series

**DOI:** 10.3390/jcm14176062

**Published:** 2025-08-27

**Authors:** Magdalena Hubert, Maciej Gawęcki, Andrzej Grzybowski

**Affiliations:** 1 Department of Ophthalmology of Pomeranian Hospitals in Wejherowo, 84-200 Wejherowo, Poland; magdalenahubert111@gmail.com (M.H.); maciej@gawecki.com (M.G.); 2 Dobry Wzrok Ophthalmological Center in Gdansk, 80-392 Gdansk, Poland; 3 Department of Ophthalmology, University of Warmia and Mazury, Oczapowskiego 2, 10-719 Olsztyn, Poland; 4 Institute for Research in Ophthalmology, Foundation for Ophthalmology Development, Mickiewicza 24, 61-836 Poznan, Poland

**Keywords:** LCHADD, macular neovascularization, chorioretinopathy, anti-VEGF, inherited metabolic disorder, SD-OCT

## Abstract

**Background:** Long-chain 3-hydroxyacyl-CoA dehydrogenase deficiency (LCHADD) is a rare autosomal recessive metabolic disorder affecting long-chain fatty acid β-oxidation. A hallmark feature of LCHADD is progressive chorioretinopathy, which may lead to severe visual complications, including macular neovascularization (MNV). The goal of the study was to analyze MNV in patients with genetically confirmed LCHADD. **Methods:** Data of 8 patients with LCHADD from the Kaszubia region in Poland followed in the clinic were retrospectively analyzed. The analyses included genetic confirmation, ophthalmologic examinations, spectral-domain optical coherence tomography (SD-OCT), and treatment responses. **Results:** Two patients with MNV in the course of LCHADD were identified. In Patient 1, a 9-year-old female, unilateral MNV at the fibrotic stage with a visual acuity of counting fingers was diagnosed in the right eye. No treatment was administered. The left eye remained stable, maintaining a best corrected visual acuity (BCVA) of 0.9 on the decimal Snellen chart. Patient 2, male, was followed from age 8 to 16 and during that time developed bilateral MNV. The right eye presented with inactive MNV at the age of 9, resulting in BCVA reduction to 0.3 without active fluid, and remained stable without intervention. The left eye developed active MNV at age 15 with subretinal fluid and retinal edema. Treatment with five intravitreal injections of ranibizumab led to complete resolution and recovery of BCVA to 1.0. **Conclusions:** MNV, although rare, can develop in pediatric LCHADD patients silently and bilaterally. Early detection through regular ophthalmologic screening is crucial, as timely anti-VEGF treatment can preserve or restore vision. Delayed diagnosis may result in irreversible damage and limited therapeutic benefit.

## 1. Introduction

Long-chain 3-hydroxyacyl-CoA dehydrogenase deficiency (LCHADD) is a congenital disorder caused by a deficiency of long-chain fatty acid dehydrogenase, leading to impaired β-oxidation of long-chain fatty acids. Incidence of LCHADD in the general population is low and estimated, for example, at about 1:120,000 in Poland and at 1:250,000 according to combined data from Australia, Germany and the United States [1,2]. Incidence of this disorder may be higher in small ethnic groups with a carrier effect, as in the Pomeranian district (Poland), where it reaches a frequency of 1:16,900 [1]. The condition is usually diagnosed in childhood and confirmed through genetic testing, specifically identifying the c.1528 G>C mutation in the *HADHA* gene. LCHADD is inherited in an autosomal recessive manner, resulting in a higher prevalence within small or isolated ethnic groups [3]. Clinically, LCHADD manifests with life-threatening episodes of hypoketotic hypoglycemia, particularly during periods of infection, fasting, or physical exertion. Additional findings may include cardiomyopathy with arrhythmia, recurrent rhabdomyolysis accompanied by muscular hypotonia, peripheral polyneuropathy, and liver failure. Without timely diagnosis and management with a low in-chain-fatty-acid diet along with supplementation of medium-chain triglyceride oil (MCT), the disease can be fatal [4]. The survival rate with proper care is estimated at more than 60% [5].

A hallmark feature of LCHADD is pigmentary chorioretinopathy, observed in all affected individuals. This ocular condition typically progresses to retinal atrophy over time, despite adequate systemic management [6,7]. A large percentage of patients develop progressive myopia, sometimes accompanied by scleral staphyloma. A rare but vision-threatening complication is macular neovascularization (MNV), with only a few reported cases in the literature to date [8,9].

In this paper, we evaluated retrospectively the electronic documentation of our LCHADD patients at our clinic to analyze the history of MNV. Within the studied group of 8 patients (16 eyes), we identified three episodes of macular neovascularization occurring in two children originating from the Kasubia region in Poland. In both patients, the diagnosis was established shortly after birth through genetic testing.

## 2. Material and Methods

The medical records of eight patients with LCHADD, followed long term at the Department of Ophthalmology, Pomeranian Hospital in Wejherowo, were reviewed for the occurrence of MNV. MNV was diagnosed in three eyes of two patients during the course of the disease.

All patients underwent planned annual follow-up visits, which included a comprehensive ophthalmological evaluation consisting of: best-corrected visual acuity (BCVA) testing using the decimal Snellen chart; anterior and posterior segment examination with a slit lamp; color fundus photography (Visucam NM/FA, Carl Zeiss Meditec AG, Jena, Germany, 2010; Optopol Revo FC, Optopol Technology, Zawiercie, Poland, 2022); intraocular pressure measurement; and spectral-domain optical coherence tomography (SD-OCT) scanning (Zeiss Cirrus 5000 Angioplex, Carl Zeiss Meditec AG, Jena, Germany, 2017; Optopol Revo FC, Optopol Technology, Zawiercie, Poland, 2022). Protocols used for SD-OCT measurements were standard macular cube 512 × 128 for Zeiss Angioplex and 6 × 6 mm for Optopol Revo FC.

Optical coherence tomography angiography (OCTA) was performed in selected cases after the device became available (Optopol Revo FC, Optopol Technology, Zawiercie, Poland, 2022). Protocol 6 × 6 mm was used for MNV evaluation. Following the detection of MNV, patients were monitored more frequently, depending on MNV activity, and were administered treatment. Eyes with inactive MNV were examined twice yearly, while treated eyes were evaluated monthly after the administration of intravitreal medication.

## 3. Patient 1

**Figure 1:**
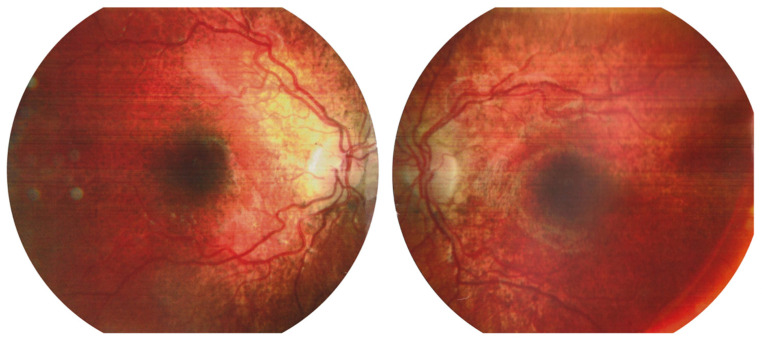
Images of the fundus of the left and right eyes at the beginning of the follow-up. Retinal thinning with salt-and-pepper-like pattern is detected at the periphery.

The first case involved a 9-year-old female patient who had been under ophthalmological follow-up for three years. At baseline, the patient’s BCVA was 1.0 (Snellen) in both eyes, with a mild hypermetropic correction of +1.0/−0.50 × 180°. During the initial years of observation, typical signs of progressive chorioretinopathy were noted in the fundus of both eyes. Baseline fundus imaging revealed chorioretinal atrophy extending from the macular center toward the equator, along with minor pigment clumping in the foveal region (Figure 1).

**Figure 2:**
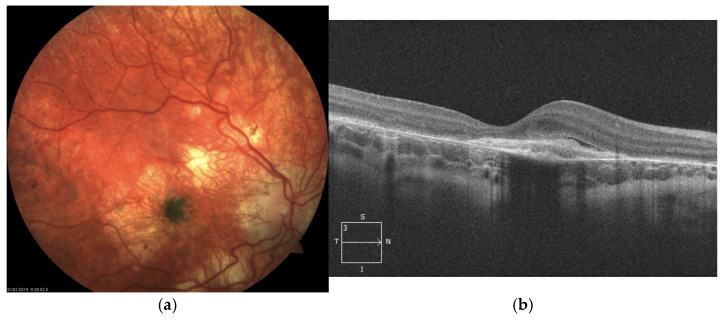
Right eye of the patient 1. Significant chorioretinal atrophy is visible in the posterior pole with a large pigment clump in the center (**a**). SD-OCT scan reveals hyperreflective structure at the level of and beneath the RPE referring to subretinal fibrosis following MNV (**b**).

The atrophy progressed with increasing pigmentary changes in the macular center. At one point during follow-up, when the patient was 9 years old, significant dense pigment concentration was observed in the right eye, accompanied by the presence of subretinal fibrosis and subretinal fluid (SRF), as detected on SD-OCT (Figure 2). At that time, BCVA in the right eye had deteriorated to counting fingers, while the left eye maintained a BCVA of 1.0.

SD-OCT scans presented hyperreflective material at the level of the RPE with a minor amount of subretinal fluid. Taking into account very low visual acuity and such presentation, the diagnosis of inactive MNV in the right eye was made. Hence, no treatment was administered.

**Figure 3:**
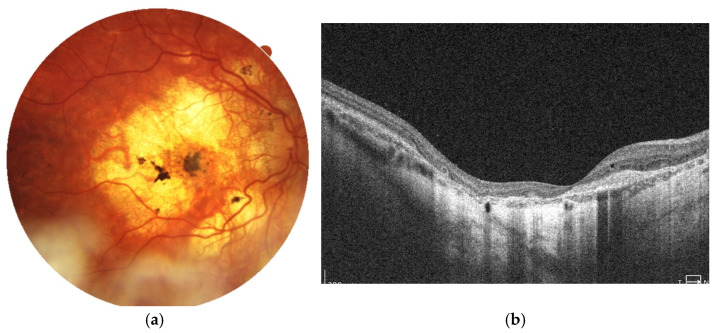
Images of the right eye: (**a**) color fundus photograph and (**b**) SD-OCT scan. Marked chorioretinal atrophy is visible in the posterior pole, accompanied by a large pigment clump consistent with macular neovascularization (MNV) scarring. The SD-OCT reveals retinal thinning and hyperreflective material at the level of the retinal pigment epithelium (RPE), corresponding to fibrotic tissue. No signs of active MNV are observed.at the level of the RPE corresponding to fibrosis.

The latest fundus images and SD-OCT scans of the right eye are shown in Figure 3a,b. The images demonstrate progressive atrophy of the retinal pigment epithelium (RPE) and the choroid in the posterior pole followed by development of staphyloma. Pigmentary changes following MNV remain stable.

**Figure 4:**
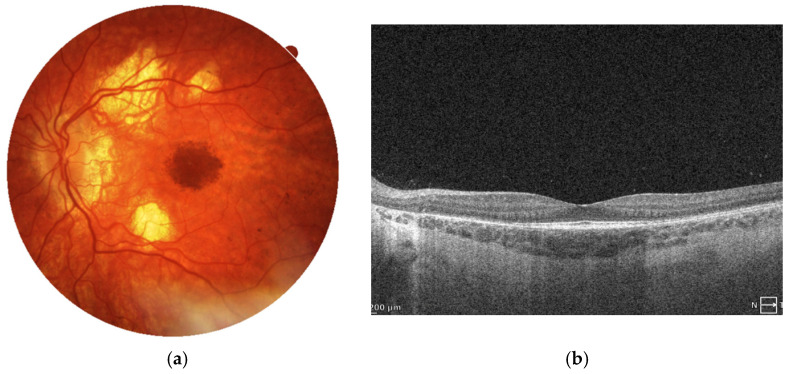
Current images of the left eye of Patient 1. (**a**) The color fundus photograph shows retinal thinning and chorioretinal atrophy predominantly along the vascular arcades, with marked hyperpigmentation in the foveal region. (**b**) The SD-OCT scan reveals relatively preserved retinal architecture, with its generalized thinning. There is a marked loss of the outer retinal layers, especially the ellipsoid zone. This symptom results clinically in the loss of contrast sensitivity despite preservation of visual acuity.

BCVA in the right eye remains at the level of counting fingers and moderate myopia appears in autorefractometry, while the left eye maintains a high visual acuity of 0.9 with a mild myopic correction of −0.25 D. The left eye also exhibits chorioretinal atrophy though in lesser extent compared to the right eye and with relatively mild central pigmentary changes. SD-OCT scan presents relatively normal retinal architecture with moderate reduction in its thickness. Figure 4a,b.

## 4. Patient 2

The second patient has been under ophthalmologic follow-up from the age of 8 until the present age of 16. The follow-up visits were scheduled once a year before the diagnosis of MNV. At the beginning of follow-up, the patient had full best-corrected visual acuity in both eyes (1.0), with mild myopia in the right eye (−0.50 D), pigmentary changes in both foveas, and peripheral pigmentary alterations resembling a “salt-and-pepper” pattern.

**Figure 5:**
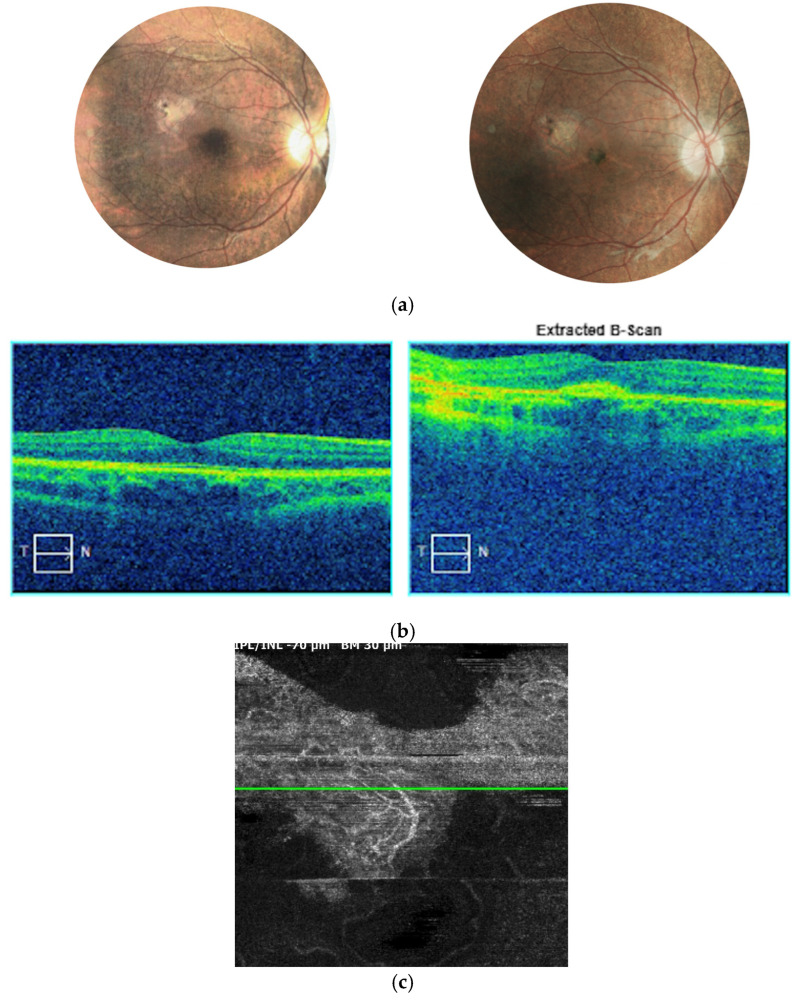
Color fundus photographs (**a**) depict the fundus appearance before and at the time of MNV diagnosis. Retinal thinning is visible in both images, while the second image additionally shows focal hyperpigmentation corresponding to fibrotic MNV. SD-OCT scans (**b**) illustrate the progression from relatively preserved retinal architecture (left image) to the development of a hyperreflective fibrotic scar at the level of the retinal pigment epithelium. Angio-OCT scan (**c**) at the avascular level of the retina presents inactive MNV with just large vessel channels noted within the subretinal scar.

At the age of 9, a significant decrease in BCVA was observed in the right eye, declining to 0.3 on the Snellen scale. Fundus examination revealed a pigment clump within the foveal region, while SD-OCT showed a hyperreflective structure at the level of the RPE. The high reflectivity of the lesion, along with the absence of intraretinal or subretinal fluid, suggested the presence of inactive MNV in a cicatricial stage—the finding was confirmed by angio-OCT scan, where at the avascular level, well organized, inactive MNV was visualized. With such characteristics, MNV was not treated with anti-VEGF medication. Comparative changes in the right eye, documented by color fundus photography and SD-OCT imaging, are presented in Figure 5a,b.

**Figure 6:**
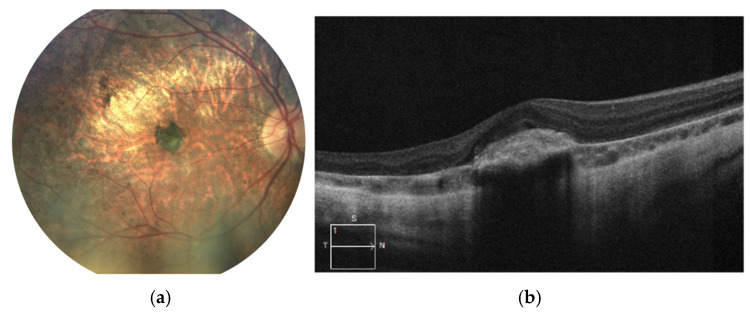
Right eye of Patient 2 at final follow-up. The color fundus photograph (**a**) shows chorioretinal atrophy and pigment clumping along the fibrotic scar following MNV. The SD-OCT image (**b**) reveals a hyperreflective scar at the level of the RPE, accompanied by neurodegenerative changes in the outer neurosensory retina.

Over the following years of observation, pigment concentration in the macular center and chorioretinal atrophy enlarged and myopia increased to the level of −5.0 D. No signs of MNV activity were detected on SD-OCT scans at any time point (Figure 6a,b). The patient remains under observation of the clinic with actual BCVA at 0.2 Snellen.

**Figure 7:**
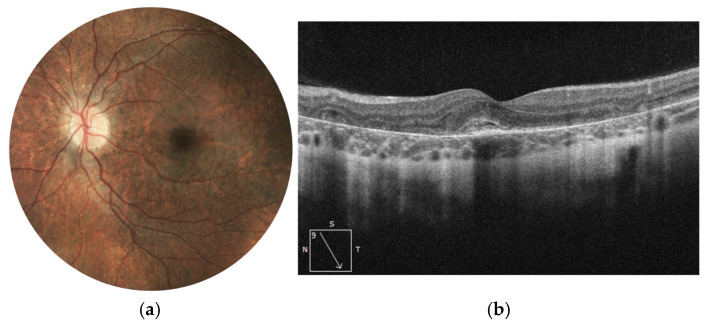
Patient 2 left eye before the onset of MNV (color fundus photograph) and at the diagnosis of MNV (SD-OCT scan). Retinal thinning is visible at the fundus without specific focal lesions (**a**). SD-OCT scan shows hyperreflective mass of MNV with small amount of subretinal fluid above the lesion (**b**).

**Figure 8:**
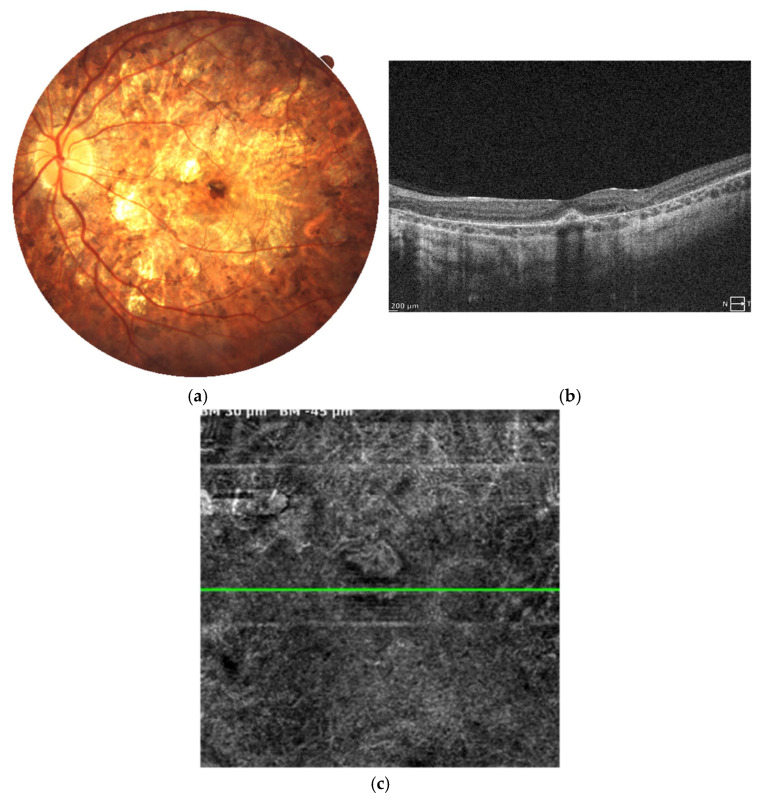
Left eye of patient 2 a few months after anti-VEGF treatment. Fundus of the eye presents with significant atrophic alterations in the posterior pole, which reflects the natural course of disease progression. (**a**) A small pigment clump refers to the inactive MNV in the foveal center. SD-OCT scan (**b**) reveals a hyperreflective spot at the level of the RPE and beneath, referring to scarring post MNV. No subretinal fluid or retinal edema is detected. Angio-OCT scan (**c**) at the level of choriocapillaris shows the inactive MNV without small sprouting capillary vessels.

MNV occurred in the same patient in the left eye as well. At the age of 15, the patient complained of sudden visual acuity decline and was promptly examined in the clinic. A small retinal hemorrhage was observed at the center of the fundus, with a moderate decline of BCVA to the level of 0.63 Snellen (with myopic correction of −2.75 Dsph). SD-OCT examination revealed a hyperreflective mass in the macular center at the level of the RPE, accompanied by subretinal fluid and neurosensory retina edema (Figure 7a,b). Anti-VEGF treatment was administered immediately (ranibizumab–Lucentis) in a pro re nata fashion. A total of five intravitreal injections were performed to achieve complete regression of MNV activity, as evaluated by SD-OCT and OCT angiography (OCTA) scans (Figure 8a–c). BCVA improved to a normal level of 1.0 with −3.0 D correction. The patient remains under close follow-up in our clinic.

Summary of specific ophthalmic findings of the two patients is presented in Table 1. Evolution of BCVA in MNV eyes is presented in Table 2.

## 5. Discussion

Macular neovascularization in LCHADD has been reported so far in only two studies [6,7]. The cases presented in our study involve pediatric patients, which have implications for early diagnosis and management. Young children are less likely to report decreased visual acuity, making regular ophthalmic examinations essential for early MNV detection in LCHADD.

In our case series, two out of three MNV incidents were diagnosed at the stage of subretinal fibrosis during routine follow-up visits. In such advanced cases, anti-VEGF treatment is ineffective and therefore was not administered. Conversely, one patient who reported visual decline was promptly treated with a series of intravitreal injections and achieved excellent visual acuity of 1.0 on the Snellen chart. This highlights the importance of early detection and timely intervention in managing LCHADD-associated MNV.

Notably, two of the MNVs were missed during their active phase despite close monitoring and regular yearly follow-up visits. This suggests that MNVs in LCHADD may generally have low proliferative potential but a strong tendency toward rapid fibrosis, which likely developed between scheduled examinations. The third case, which was successfully treated, demonstrates the potential for substantial visual recovery when intervention is initiated early.

Wongchaisuwat et al. reported a larger case series involving nine MNVs in seven LCHADD patients aged six to 36 years [9]. None of the MNVs reported in this study were noted in a child. The authors estimated MNV incidents prevalence of 21% in LCHADD but noted that this may be an underestimate, as with introduction of highly sensitive OCTA in screening for MNV, this number might be higher. In our group of patients with LCHADD followed long term, MNV occurred in three out of 16 eyes (two of eight patients); thus, the percentage is similar [10]. Sacconi et al. presented just one case of unilateral inactive MNV in a female adult with LCHADD [8]. The condition was diagnosed at cicatrical stage and a low BCVA of 20/1000.

Also, similarly to our findings, some cases in their study were subclinical until detected later with OCTA. Six of their patients did not report any vision loss during MNV development, consistent with our observations. In our study, Patient 2—who did report vision decline—already had significant visual impairment in the contralateral eye, which may have contributed to their awareness of symptoms. In cases with unilateral involvement, symptoms might go unnoticed by the patient.

SD-OCT imaging of the active neovascular membrane in our case showed a pattern similar to that seen in myopic MNV, with limited disruption of the neurosensory retina. The membrane appeared compact and was located above the RPE, consistent with type 2 MNV. At the later fibrotic stage, MNV in our patients was associated with the presence of dense pigmentation. These morphological findings are consistent with the observations reported by Wongchaisuwat et al. and Sacconi et al. [8,9]. The advent of OCT angiography has significantly improved the ability to detect silent or subclinical MNVs in patients with LCHADD. This is particularly important, as traditional angiographic methods used for its diagnosis are not always reliable. In inherited retinal diseases, the angiographic signs of MNV are often masked by chorioretinal atrophy and fibrosis. Since OCTA directly visualizes the vascular network, it appears to be more effective in detecting MNV in such cases. In our study, OCTA was available for the third MNV case, which was successfully treated. Wongchaisuwat et al. employed this imaging modality in a larger case series, demonstrating its high sensitivity for MNV detection [9].

The pathogenesis of LCHADD-related chorioretinopathy and its complications, including MNV, remains unclear. Chorioretinal atrophy, accompanied by substantial loss of choroidal tissue, leads to hypoxia and subsequent upregulation of angiogenic factors. It is also associated with marked retinal thinning and the formation of cracks in Bruch’s membrane, which trigger inflammatory responses and further expression of growth factors [11,12]. These mechanisms have also been implicated in the development of myopic neovascularization (MNV), a complication frequently observed in patients with LCHADD. In LCHADD, MNV has been reported almost exclusively in the fovea—a distribution similarly characteristic of myopia-related MNV. This central predilection may be explained by the fact that the greatest proportional loss of choroidal vascularity, which promotes angiogenesis, occurs in the thickest portion of the choroid, located centrally. Despite these similarities, MNV in LCHADD can also develop in patients with emmetropia or low-grade myopia, indicating that the overlap between these conditions is only partial. In LCHADD, the loss of retinal and choroidal cells is likely more extensive, which may account for the higher incidence of MNV—estimated at approximately 20%—compared with 5–11% in the myopic population [13]. It is plausible that choroidal hypoxia associated with both myopia and LCHADD acts synergistically to promote the development of this complication. Some researchers have suggested that poor metabolic control and chronically elevated serum levels of long-chain fatty acids, specifically long-chain 3-hydroxyacylcarnitines, may contribute to disease progression and vision loss [14,15,16]. However, no direct relationship has been established between these metabolic factors and the occurrence of MNV in LCHADD.

### Study Limitations

We acknowledge that the main limitation of our study is the small number of cases. However, this is an inherent challenge given the extreme rarity of the disease, making it difficult to assemble larger cohorts. Consequently, conclusions and therapeutic decisions must rely on the current general knowledge and available case reports. A possible bias in estimating the incidence of MNV in LCHADD may also stem from the composition of our study cohort, which originated from the Pomeranian region of Kasubia. This, however, reflects the real-world distribution of LCHADD, which is often confined to small ethnic groups with a genetic predisposition. As such, this type of bias is difficult to avoid.

## 6. Conclusions

MNV can develop in pediatric LCHADD patients silently and bilaterally. Early detection through regular ophthalmologic screening is crucial, as timely anti-VEGF treatment can preserve or restore vision. Available data from published reports suggest OCTA as the most sensitive tool for MNV detection in LCHADD patients. Delayed diagnosis may result in irreversible damage and limited therapeutic benefit.

## Figures and Tables

**Table 1:** Clinical characteristics of patients with macular neovascularization in LCHADD.

Parameter	Patient 1 RE	Patient 2 RE	Patient 2 LE
Age at MNV diagnosis	8.5	8.5	15
BCVA of MNV eye upon presentation (Snellen decimal)	CF	0.3	0.6
BCVA of the fellow eye at diagnosis (Snellen decimal)	1.0	1.0	0.2
Subjective complaints	none	none	dense central scotoma
SD-OCT findings at presentation	subretinal fibrosis, minor amount of SRF, chorioretinal atrophy	subretinal fibrosis, minor chorioretinal atrophy	subretinal fibrosis, minor amount of SRF, chorioretinal atrophy
Fundoscopy findings	dense pigment concentration in the fovea, surrounded by chorioretinal atrophy	dense pigment concentration in the fovea	small foveal hemorrhage
Treatment	none	none	6× intravitreal ranibizumab
Final BCVA of MNV eye (Snellen decimal)	CF	0.2	1.0

BCVA—best corrected visual acuity, RE—right eye, LE—left eye, SD-OCT—spectra domain optical coherence tomography, CF—counting fingers.

**Table 2:** Changes in BCVA over time in LCHADD patients with MNV (Snellen decimal scale).

Age (Years)	6	7	8	9	10	11	12	13	14	15	16	17
Patient 1 RE	1.0	1.0	1.0	MNV 0.3	0.01	0.02	0.02	0.02	0.2	0.06	0.06	0.06
Patient 2 RE	N/A	N/A	1.0	MNV 0.3	0.3	0.3	0.1	0.2	0.2	0.1	0.2	0.16
Patient 2 LE	N/A	N/A	1.0	1.0	1.0	1.0	1.0	1.0	0.9	MNV 0.2 ≫ 6 × ranibizumab ≫ 1.0	1.0	1.0

RE—right eye, LE—left eye, MNV—macular neovascularization, BCVA—best corrected visual acuity, LCHADD—long-chain 3-hydroxyacyl-CoA dehydrogenase deficiency.

## Data Availability

All available data are contained within the manuscript.
